# The influence of proton pump inhibitors on tissue attachment around teeth and dental implants: A scoping review

**DOI:** 10.1002/cre2.616

**Published:** 2022-07-07

**Authors:** Bhavneet K. Chawla, Robert E. Cohen, Elizabeth M. Stellrecht, Lisa M. Yerke

**Affiliations:** ^1^ Department of Periodontics and Endodontics, School of Dental Medicine, University at Buffalo The State University of New York Buffalo New York USA; ^2^ University Libraries, University at Buffalo The State University of New York Buffalo New York USA

**Keywords:** dental implants, periodontal disease, periodontitis, proton pump inhibitors

## Abstract

**Objectives:**

Proton pump inhibitors, such as omeprazole and pantoprazole, are frequently prescribed for the treatment of acid reflux. However, those medications have been shown to affect a variety of physiologic processes, including bone homeostasis and the gastrointestinal microbiome. The objective of this study was to assess the relationship between proton pump inhibitors and attachment levels around teeth and dental implants. A scoping review was performed to assess the extent and quality of the relevant literature.

**Materials and Methods:**

We used the Preferred Reporting Items for Systematic Reviews and Meta‐Analyses extension for scoping reviews (PRISMA‐ScR) and searched four relevant biomedical literature databases in addition to the grey literature. Keywords in the title and abstract fields, and subject headings for proton pump inhibitors, teeth, and dental implants were included as search terms.

**Results:**

Overall search results identified 791 publications which, after applying the inclusion and exclusion criteria, yielded 27 publications that were further analyzed for relevance and quality of scientific evidence. The majority of eligible publications were retrospective cohort studies. Following critical analysis, 13 publications, including six abstracts, were used to assess the effect of proton pump inhibitors on tissue attachment around teeth and dental implants.

**Conclusions:**

There are few high‐quality studies describing the effect of proton pump inhibitors on tissue attachment around teeth and dental implants. Nevertheless, among the included papers with the fewest confounding factors, there was a positive relationship between proton pump inhibitors and soft tissue attachment levels around teeth, and a predominantly negative but variable effect of proton pump inhibitors on the bone level around dental implants. Additional well‐controlled prospective studies are required to fully elucidate those relationships.

## 1 INTRODUCTION

Proton pump inhibitors (PPIs) suppress gastric acidity by inhibiting the H^+^/K^+^ ATPase proton pump in parietal cells (Al Subaie et al., 2016). PPIs, such as pantoprazole and omeprazole, are used for the prevention and treatment of acid‐related conditions including esophageal, stomach, and duodenal ulcers; ulcers associated with the use of nonsteroidal anti‐inflammatory drugs (NSAIDs); gastroesophageal reflux disease (GERD); and Zollinger–Ellison syndrome (Aghaloo et al., 2019). PPIs also are used in combination with antibiotics for eradicating *Helicobacter pylori* which, in conjunction with gastric acid, causes ulcers of the stomach and duodenum (Aghaloo et al., 2019). In the United States, PPIs typically are ranked among the top 10 most frequently prescribed medications (Fuentes et al., 2018), with the use of PPIs in outpatient care doubling from 3.9% in 1999 to 7.8% in 2012 (Freedberg et al., 2017). PPIs at lower doses also are available in over‐the‐counter formulations for the treatment of acid reflux (Targownik et al., 2008).

The primary physiologic change induced by PPI therapy is a profound suppression of gastric acid secretion, leading to impaired nutrient absorption (Yang, 2012). Since dietary calcium is consumed in the form of insoluble calcium salts, the release of soluble calcium depends on the acidic environment of the gastrointestinal (GI) tract (Sipponen & Härkönen, 2010). However, decreased gastric acid production leads to less effective calcium dissolution and ionization, which frequently results in correspondingly less calcium absorption in the proximal small intestine (Sipponen & Härkönen, 2010). Due to the decrease in gastric acidity from the pharmacologic effect of PPIs, calcium absorption declines, which potentially affects osteoclast function and leads to a decrease in bone mineral density (Johnson, 2016; Khalili et al., 2012).

In contrast, there is evidence that PPIs also have bone protective properties. PPIs have a direct effect on osteoclasts by inhibiting osteoclast‐mediated bone remodeling. Osteoclasts contain proton pumps that can be inhibited by PPIs and alter osteoclast‐mediated bone resorption (Jo et al., 2015; Wu et al., 2017). The PPI pantoprazole also was found to indirectly decrease bone resorption both in vivo and in vitro by inhibiting a specific pathway for osteoclastogenesis (Li et al., 2020). The PPI omeprazole has been shown to decrease Ca^2+^ release from mouse calvaria (David & Baron, 1995), as well as decrease urinary secretion of Ca^2+^ in humans, further supporting inhibition of bone resorption by a direct effect on osteoclast function (David & Baron, 1995; Targownik & Leslie, 2011). Moreover, in an in vitro study, Costa‐Rodrigues et al. (2013) proposed that PPIs have direct deleterious effects on osteoblasts and osteoclasts, decreasing bone turnover.

Gastric acid suppression induced by PPI therapy results in hypergastrinemia, increased production of the peptide hormone gastrin (Dacha et al., 2015; Yang, 2012). Hypergastrinemia, in turn, has a stimulatory effect on the parathyroid glands, in addition to trophic effects on gastrointestinal tissues (Yang, 2012). Parathyroid hormone (PTH) is the principal calcium‐regulating hormone and plays a pivotal role in calcium and bone metabolism. As the primary calciotropic hormone, PTH maintains serum calcium concentrations by stimulating bone resorption, increasing renal tubular calcium reabsorption, and stimulating renal calcitriol production, all which lead to increased active transport of calcium in the proximal small intestine (Sipponen & Härkönen, 2010; Yang, 2012). Persistently elevated PTH secretion, in relation to calcium serum concentration, might result in loss of bone strength and quality. Consequently, both hypergastrinemia and calcium malabsorption have the potential to negatively influence bone and mineral metabolism, at least partially through the induction of hyperparathyroidism (Yang, 2012).

Reduced stomach pH diminishes the gastric acid barrier, allowing pathogenic microbial species to survive (Mishiro et al., 2018). Accordingly, PPIs have been shown to modify the host microbiota in the GI tract and can induce dysbiosis which, in turn, can facilitate the onset of certain GI disorders (Bruno et al., 2019). Chronic PPI use has been shown to decrease *Bacteroides* and increase *Firmicutes* in the GI tract, as well as act as a risk factor for *Clostridium difficile* infection (CDI) (Singh et al., 2018). The risk of CDI has been reported to increase when PPIs are used with antibiotics (Singh et al., 2018).

Current evidence also suggests that changes in intestinal microbiota might increase the risk of infection (Jackson et al., 2016), and bacterial overgrowth has been observed in the small intestine in conjunction with the use of PPIs (Singh et al., 2018). PPIs prescribed for the treatment of GERD have been reported to significantly increase salivary pH, potentially affecting the oral microbiome (Mishiro et al., 2018). It has been reported that PPI users harbor increased numbers of oral microbiome species such as *Rothia dentocariosa, R. mucilaginosa*, the genera *Scardovia* and *Actinomyces*, and the family Micrococcaceae in their gut microbiome (Imhann et al., 2016).

The effect of PPIs on bone metabolism most likely is multifactorial. Increased duration of drug use, greater dosages, and age in excess of 60 years, all are associated with an increased risk of osteoporosis‐related fractures (Jo et al., 2015; Targownik et al., 2008). The existence of multiple potential mechanisms through which PPIs might affect bone metabolism, in combination with the numerous confounders that might exist or await identification, might explain in part why other studies (including a recent systematic review) have not demonstrated a relationship between PPI and loss of bone mineral density (Aleraij et al., 2020; Targownik et al., 2008, 2010).

Periodontitis is characterized by microbially‐associated, host‐mediated inflammation that results in loss of periodontal attachment (Tonetti et al., 2018). Periodontal pathogenesis is attributed primarily to the host response toward microbial plaque. Once immunoinflammatory processes begin, the connective tissue attachment and alveolar bone are destroyed (Hienz et al., 2015). Numerous systemic conditions have been associated with loss of periodontal attachment, including Down syndrome, epidermolysis bullosa, systemic lupus erythematosus, diabetes mellitus, obesity, osteoporosis, arthritis, and smoking (Albandar et al., 2018). Risk factors for periodontitis also include cancer chemotherapy medications, vascular endothelial growth factor inhibitors, and tyrosine kinase inhibitors (Albandar et al., 2018). Since PPIs are known to affect bone mineral metabolism, it is possible that the use of those medications also might affect alveolar bone. However, the potential effects of PPIs on periodontal health remain to be elucidated.

Similarly, dental implants have become a reliable solution for the functional and esthetic rehabilitation of partially and completely edentulous patients. Osseointegration is necessary for implant survival (Mangano et al., 2016). Factors proposed to affect osseointegration include age, smoking, periodontitis, diabetes, head, and neck radiation, and post‐menopausal estrogen therapy, as well as some systemic medications such as anti‐hypertensives, selective serotonin reuptake inhibitors (SSRIs), and PPIs (Aghaloo et al., 2019; Koldsland et al., 2009; Moy et al., 2005; Wu et al., 2017). Indeed, recent studies have suggested an increased risk of dental implant failure among patients taking PPIs, compared to patients not using those medications (Aghaloo et al., 2019; Al Subaie et al., 2016; Chrcanovic et al., 2017). However, the effects of PPIs on the bone supporting dental implants have not previously been described. Therefore, we sought to investigate the potential effects of PPIs on the periodontium, and recently reported on the relationship between attachment levels around teeth (Herrmann et al., 2022a; Yerke & Cohen, 2019; Yerke et al., 2019), and bone levels around dental implants (Ursomanno et al., 2019, 2020). Those studies led us to perform a scoping review to critically assess the existing literature. Consequently, the objective of this review was to evaluate the quantity and quality of the evidence describing the relationship between PPIs and tissue attachment around teeth and dental implants.

## 2 METHODS

This review used the Preferred Reporting Items for Systematic Reviews and Meta‐Analyses extension for scoping reviews (PRISMA‐ScR) reporting guidelines for preparation of this manuscript. An electronic search was completed between December 5 to December 7, 2019, and further updated on January 15, 2021, May 11, 2021, and May 19, 2022, using PubMed, Embase, Cochrane Central Register of Controlled Trials, and CINAHL without any date or language restrictions. The search was comprised of both subject headings, as well as keyword terms representing proton pump inhibitors and bone around teeth and dental implants. The search was created in PubMed as described in Table 1, and then translated to other databases. Since this scoping review did not involve human research according to the University at Buffalo Health Sciences Institutional Review Board, it was therefore exempt from requiring IRB approval and informed consents.

**Table 1:** Search strategy for PubMed

1. “Proton Pump Inhibitors”[Mesh]
2. Proton pump inhibitor[Title/Abstract]
3. Proton pump inhibitors[Title/Abstract]
4. “Rabeprazole”[Mesh]
5. “Lansoprazole”[Mesh]
6. “Pantoprazole”[Mesh]
7. “Omeprazole”[Mesh]
8. Rabeprazole[Title/Abstract]
9. Lansoprazole[Title/Abstract]
10. Dexlansoprazole[Title/Abstract]
11. Esomeprazole[Title/Abstract]
12. Pantoprazole[Title/Abstract]
13. Omeprazole[Title/Abstract]
14. Prevpac[Title/Abstract]
15. Aciphex[Title/Abstract]
16. Prevacid[Title/Abstract]
17. Dexilant[Title/Abstract]
18. Kapidex[Title/Abstract]
19. Nexium[Title/Abstract]
20. Protonix[Title/Abstract]
21. Zegerid[Title/Abstract]
22. Prilosec[Title/Abstract]
23. Search (((((((((((((((((((((“Proton Pump Inhibitors”[Mesh]) OR proton pump
inhibitor[Title/Abstract]) OR proton pump inhibitors[Title/Abstract]) OR
“Rabeprazole”[Mesh]) OR “Lansoprazole”[Mesh]) OR “Pantoprazole”
[Mesh]) OR “Omeprazole”[Mesh]) OR rabeprazole[Title/Abstract]) OR
lansoprazole[Title/Abstract]) OR dexlansoprazole[Title/Abstract]) OR
esomeprazole[Title/Abstract]) OR pantoprazole[Title/Abstract]) OR
omeprazole[Title/Abstract]) OR prevpac[Title/Abstract]) OR
aciphex[Title/Abstract]) OR prevacid[Title/Abstract]) OR
dexilant[Title/Abstract]) OR kapidex[Title/Abstract]) OR
nexium[Title/Abstract]) OR protonix[Title/Abstract]) OR zegerid[Title/Abstract]) OR prilosec[Title/Abstract]
24. “Dental Implants”[Mesh]
25. “Dental Implantation”[Mesh]
26. “Osseointegration”[Mesh]
27. “Bone Density
28. “Bone‐Anchored Prosthesis”[Mesh]
29. “Tooth”[Mesh]
30. “Jaw”[Mesh]
31. “Bone Resorption”[Mesh]
32. “Alveolar Bone Loss”[Mesh]
33. Dental implant[Title/Abstract]
34. Dental implants[Title/Abstract]
35. Dental implantation[Title/Abstract]
36. Osseointegrat*[Title/Abstract]
37. Endosseous[Title/Abstract]
38. Jaw[Title/Abstract]
39. Alveolar bone[Title/Abstract]
40. Tooth[Title/Abstract]
41. Teeth[Title/Abstract]
42. Bone density[Title/Abstract]
43. Bone resorption[Title/Abstract]
44. “Oral Health”[Mesh]
45. Oral health[Title/Abstract]
46. Oral complication[Title/Abstract]
47. Oral complications[Title/Abstract]
48. ((((((((((((((((((((((("Dental Implants”[Mesh]) OR “Dental Implantation"
[Mesh]) OR “Osseointegration”[Mesh]) OR “Bone Density”[Mesh]) OR
“Bone‐Anchored Prosthesis”[Mesh]) OR “Tooth”[Mesh]) OR “Jaw”[Mesh])
OR “Bone Resorption”[Mesh]) OR “Alveolar Bone Loss”[Mesh]) OR dental
implant[Title/Abstract]) OR dental implants[Title/Abstract]) OR dental
implantation[Title/Abstract]) OR osseointegrat*[Title/Abstract]) OR
endosseous[Title/Abstract]) OR jaw[Title/Abstract]) OR alveolar
bone[Title/Abstract]) OR tooth[Title/Abstract]) OR teeth[Title/Abstract]) OR
bone density[Title/Abstract]) OR bone resorption[Title/Abstract]) OR “Oral
Health”[Mesh]) OR oral health[Title/Abstract]) OR oral
complication[Title/Abstract]) OR oral complications[Title/Abstract]
49. ((((((((((((((((((((((("Proton Pump Inhibitors”[Mesh]) OR proton pump
inhibitor[Title/Abstract]) OR proton pump inhibitors[Title/Abstract]) OR
“Rabeprazole”[Mesh]) OR “Lansoprazole”[Mesh]) OR “Pantoprazole"
[Mesh]) OR “Omeprazole”[Mesh]) OR rabeprazole[Title/Abstract]) OR
lansoprazole[Title/Abstract]) OR dexlansoprazole[Title/Abstract]) OR
esomeprazole[Title/Abstract]) OR pantoprazole[Title/Abstract]) OR
omeprazole[Title/Abstract]) OR prevpac[Title/Abstract]) OR
aciphex[Title/Abstract]) OR prevacid[Title/Abstract]) OR
dexilant[Title/Abstract]) OR kapidex[Title/Abstract]) OR
nexium[Title/Abstract]) OR protonix[Title/Abstract]) OR
zegerid[Title/Abstract]) OR prilosec[Title/Abstract])) AND
(((((((((((((((((((((((("Dental Implants”[Mesh]) OR “Dental Implantation”[Mesh])
OR “Osseointegration”[Mesh]) OR “Bone Density”[Mesh]) OR “Bone‐
Anchored Prosthesis”[Mesh]) OR “Tooth”[Mesh]) OR “Jaw”[Mesh]) OR
“Bone Resorption”[Mesh]) OR “Alveolar Bone Loss”[Mesh]) OR dental
implant[Title/Abstract]) OR dental implants[Title/Abstract]) OR dental
implantation[Title/Abstract]) OR osseointegrat*[Title/Abstract]) OR
endosseous[Title/Abstract]) OR jaw[Title/Abstract]) OR alveolar
bone[Title/Abstract]) OR tooth[Title/Abstract]) OR teeth[Title/Abstract]) OR
bone density[Title/Abstract]) OR bone resorption[Title/Abstract]) OR “Oral
Health”[Mesh]) OR oral health[Title/Abstract]) OR oral
complication[Title/Abstract]) OR oral complications[Title/Abstract])

To assess the grey literature, the following additional resources were searched: International Association for Dental Research (IADR) Abstract Archive (2001–present), Proquest Dissertations and Theses Global, and clinicaltrials.gov. The keyword terms, “proton pump inhibitors,” and “bone” were used for searching the grey literature. No date or language restrictions were used at that time, and a protocol was not registered for this review.

**Figure 1:**
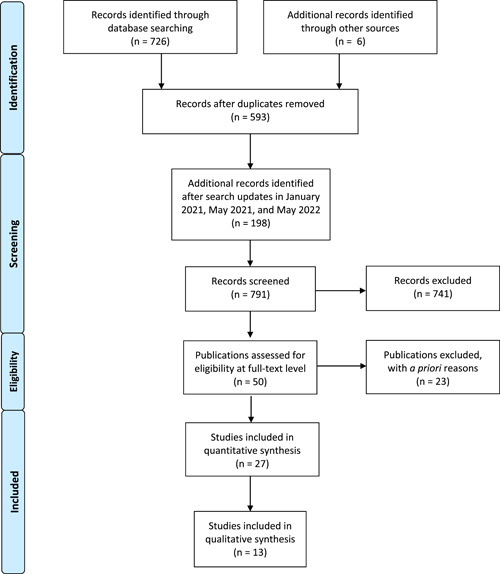
Publication selection method.

The initial search yielded 726 publications (Figure 1) through database searching, as well as an additional six publications identified through grey literature sources. The updated January 2021, May 2021, and May 2022 supplemental searches added 198 publications. After removing duplicates, 791 articles were screened by two reviewers (B.C and L.Y.) and any conflicts were resolved by a third reviewer (R.C.), resulting in the exclusion of 741 articles not meeting the inclusion criteria: proton pump inhibitors and any measurement of their effect on net bone loss/gain, bone density, oral health, periodontal health, teeth, dental implants, or osseointegration of dental implants. Although all study designs were considered, studies not performed in humans were an exclusion criterion that resulted in rejection of some publications using in vitro models that might otherwise have met the inclusion criteria. Articles with no mention of proton pump inhibitors also were excluded.

Fifty full‐text articles were further assessed for eligibility by the same reviewers. Twenty‐three articles were excluded due to outcomes (13 articles), comparators (two articles), settings (two articles), or interventions (two articles) that were not relevant to the review criteria, in addition to three papers that were excluded due to duplication, and one paper due to language other than English. After applying the inclusion, exclusion, and eligibility criteria (Table 2), a total of 27 publications were considered for further analysis.

**Table 2:** Inclusion and exclusion criteria

Inclusion:
Proton pump inhibitors (generic or brand name) and any measurement of effect on the following:
1. Bone loss/gain around teeth and/or dental implants (radiographic or otherwise) 2. Bone density around teeth and/or dental implants 3. Oral health 4. Periodontal health 5. Osseointegration of dental implants
Exclusion:
Not in English language Proton pump inhibitors absent Animal model

## 3 RESULTS

### 3.1 Aim 1

#### 3.1.1 To evaluate the *quantity* of the existing literature describing the potential relationship between PPIs and tissue attachment around teeth and dental implants: Outcome of search

There were no randomized controlled human clinical trials available that described the relationship between PPIs and tissue attachment around teeth and dental implants included in the 27 publications remaining for analysis. However, there were three systematic reviews, one retrospective record review, four retrospective cohort studies, two narrative reviews, nine abstracts, one summary of a systematic review and meta‐analysis, one mapping review, two cross‐sectional studies, one consensus report, two theses, and one commentary.

### 3.2 Aim 2

#### 3.2.1 To evaluate the *quality* of the existing literature describing a potential relationship between PPIs and tissue attachment around teeth and dental implants

All 27 publications noted in Aim 1 were critically reviewed for their ability to address the effect of proton pump inhibitors on tissue attachment around teeth or dental implants. Thirteen publications remained after critical analysis (Table 3).

**Table 3:** Summary of included publications

Authors	Publication year	Study type	Study characteristics	PPI effect on tissue attachment	Summary
Wu et al. (2017)	2017	Retrospective cohort (dental implants)	Total number of dental implants = 1773 Total number of patients = 799 Follow‐up period = 16 months Average age = 56 years	Bone loss	The percentage of implant failure was 6.8% & 3.2% among PPI users & nonusers, respectively.
Chrcanovic et al. (2017)	2017	Retrospective cohort (dental implants)	Total number of dental implants = 3559 Total number of patients = 999 Follow‐up period = 88–95 months Average age = 60 years	Bone loss	PPIs might be associated with an increased risk of dental implant failure.
Altay et al. (2019)	2019	Retrospective cohort (dental implants)	Total number of dental implants = 1918 Total number of patients = 592 Follow‐up period = 29 months Average age = 50 years	Bone loss	Implant failure among PPI users was 4.6‐fold greater.
Ursomanno et al. (2020)	2020	Retrospective chart review (dental implants)	Total number of dental implants = 1480 Total number of patients = 635 Follow‐up period = not reported Average age = not reported	Bone loss	PPIs were associated with greater bone loss around dental implants.
Romandini et al. (2021)	2021	Cross‐sectional (dental implants)	Total number of dental implants = 485 Total number of patients = 99 Follow‐up period = 93.6 months (estimated) Average age = 64 years	Less bone loss	For loaded implants and surviving for greater than 1 year, PPIs were associated with a decreased risk of dental implant failure.
Chawla et al. (2022)	2022	Retrospective chart review (teeth)	Total # of patients = 1093 Follow‐up period = not reported Average age = 61.26 years for PPI users, 55.73 years for non‐PPI users that do not smoke or have diabetes	Increase of soft tissue attachment	PPIs were inversely associated with periodontal disease severity, even when excluding for risk factors such as smoking, diabetes, systemic conditions and certain medications.
Yerke et al. (2019)	2019	AbstractRetrospective chart review (teeth)	Total number of patients = 518 Follow‐up period = not reported Average age = not reported	Increase of soft tissue attachment	PPIs were inversely associated with periodontal disease severity.
Yerke & Cohen (2019)	2019	AbstractRetrospective chart review (teeth)	Total number of patients = 518 Follow‐up period = not reported Average age = not reported	Increase of soft tissue attachment	PPIs were inversely associated with periodontal disease severity.
(Ursomanno et al. (2019)	2019	Abstract Retrospectivechart review (dental implants)	Total number of dental implants = 1480 Total number of patients = 635 Follow‐up period = unavailable Average age = not reported	Bone loss	PPI use is associated with greater bone loss around dental implants.
Yerke et al. (2021)	2021	Abstract Retrospectivechart review (dental implants)	Total number of patients = 1513 Follow‐up period = unavailable Average age = not reported	Increase of soft tissue attachment	PPIs were inversely associated with periodontal disease severity
Herrmann et al. (2022)	2022	Abstract Retrospectivechart review (dental implants)	Total number of patients = 1017 Follow‐up period = unavailable Average age = not reported	Increase of soft tissue attachment	PPIs were inversely associated with periodontal disease severity
Rogoszinski et al. (2020)	2020	AbstractRetrospective cohort (dental implants)	Total number of dental implants = 881 Total number of patients = 284 Follow‐up period = 5 years Average age = not reported	Decreased risk of peri‐implantitis as determined by radiographic and soft tissue measurements	For implants surviving 5 or more years, there was a decreased risk of peri‐implantitis among PPI users.
Rogoszinski et al. (2022)	2022	Retrospective cohort (dental implants)	Total number of patients – 284 Follow‐up period = 5 years Average age = not reported, but 66.4% were ≥ 60 years old	Long‐term implant failure, as defined by mobility, radiographic evidence, or lack of osseointegration. Peri‐implantitis as determined by radiographic crestal bone levels in conjunction with soft tissue measurements	For implants surviving 5 or more years, PPI use did not affect long‐term implant failure of peri‐implantitis after controlling for potential confounders.

### 3.3 Publications excluded from the qualitative analysis (Aim 2) of the scoping review

Two excluded publications consisted of a systematic review (Aghaloo et al., 2019), and a systematic review with meta‐analysis (Chappuis et al., 2018), that referred to articles that were separately described in the present analysis. The primary aim of the systematic review by Aghaloo et al. was to review the effects of systemic diseases and medications on implant osseointegration. The primary aim for the systematic review and meta‐analysis by Chappuis et al. was to review medication‐related dental implant failure.

A summary of a systematic review and meta‐analysis (Cheng, 2020) and an abstract (Chappuis, 2019) on the impact of medications on dental implant failure, also were excluded from the present review as those publications only summarized the review by Chappuis et al. (2018) that was itself excluded as described above.

A systematic review and meta‐analysis (Vinnakota & Kamatham, 2020) were excluded from the present study as the authors included three retrospective studies (Altay et al., 2019; Chrcanovic et al., 2017; Wu et al., 2017) that were individually critiqued in the present study.

A review by Mahri et al. (2021) was excluded from the present analysis. The primary aim of that paper was to systematically map osseointegration pharmacology using artificial intelligence. The authors identified three articles that associated PPIs with a high risk of implant failure, two of which were already individually considered in the present analysis. The third study did not meet the inclusion criteria as it measured the effect of postoperative systemic administration of omeprazole on bone healing and implant osseointegration in rat tibiae versus alveolar bone (Al Subaie et al., 2016).

A review article (Surabhi, 2019) describing the effect of medications on dental prostheses was excluded. The primary aim of that study was to review the association between multiple systemic medications and dental implants. The author did not indicate which component studies were considered to conclude that PPIs alter the success of implant therapy. In fact, there was no mention of any study that was reviewed to make any conclusions.

A narrative review (Mester et al., 2019) describing the impact of proton pump inhibitors on bone regeneration and implant osseointegration also was excluded from further analysis. The primary aim of that study was to assess the effect of PPIs on dental and orthopedic implants. The orthopedic implant component did not meet the inclusion criteria of the present review, and the dental implant component included two retrospective cohort studies that already met the study criteria and were individually evaluated in Aim 1 and 2 (Chrcanovic et al., 2017; Wu et al., 2017).

A consensus report by Jung et al. (2018), describing the influence of implant length, design, and medications on clinical and patient‐reported implant outcomes also was excluded. In that report, there was only one reference to the effect of medications on dental implants, the systematic review and meta‐analysis by Chappuis et al. (2018), previously discussed, in which PPIs were included among the medications that were considered. Consequently, the report by Jung et al. was excluded from the qualitative analysis since the component studies were individually analyzed in the present review.

An invited commentary (Tamimi & Wu, 2017) on osseointegration pharmacology was excluded. The authors discussed four groups of drugs that could affect osseointegration. One of the studies referenced in the commentary did not meet the inclusion criteria of the present review because the authors measured only the effect of limited postoperative systemic administration of omeprazole on bone healing and implant osseointegration in rat tibiae, not in alveolar bone (Al Subaie et al., 2016). Another study included in that commentary already was considered in the present study (Wu et al., 2017). Consequently, the invited commentary was excluded, and the component study that met the inclusion criteria was separately considered. Those authors also produced a thesis (Wu, 2016) that met the inclusion criteria of the present scoping review. However, the thesis subsequently was excluded from further qualitative analysis since the section pertinent to PPIs and periodontal tissue attachment was later published (Wu et al., 2017) and separately included in our review.

An abstract by Nag (2018), which summarized a scoping review on the influence of PPIs on dental implant failure, was excluded from Aim 2. The extent to which the publication addressed the inclusion and exclusion criteria of this study could not be independently ascertained, and the authors did not reference the component studies that were included. A full‐length follow‐up manuscript never was published.

An abstract of the 2021 review by Mahri and Tamimi (2019) that was previously discussed in the current review, was excluded from the present study. In this abstract, the authors evaluated 247 publications. The primary objective was to assess the effect of various medications on bone‐implant osseointegration, with PPIs as one of those drugs. The abstract did not describe the studies or various medications that were evaluated in their review, making it unclear if it met the inclusion criteria of the present study.

A 2021 thesis (Chawla & Yerke, 2021) that was comprised of two subsequently submitted manuscripts was excluded from the qualitative analysis: one manuscript was a preliminary version of this scoping review. The second manuscript was a cross‐sectional study published after completion of the thesis (Chawla et al., 2022), and is critiqued separately in the qualitative analysis, below.

### 3.4 Publications included in the qualitative analysis of the scoping review

Wu et al. (2017), performed a retrospective cohort study that included a total of 1773 osseointegrated dental implants in 799 patients (133 implants in 58 PPI users and 1640 in 741 nonusers) who were treated between January 2007 and September 2015. The failure rate among PPI users was 6.8%, and 3.2% in nonusers, supporting the authors' conclusions that PPI use is associated with a higher rate of dental implant failure. The strengths of this study included large sample size, the use of implants obtained from the same manufacturer, and all procedures or surgeries performed by a single clinician. Limitations included the retrospective nature of their study and the lack of statistical analysis of the differences between PPI users and nonusers at the patient level. There also was a statistically significant difference between the number of PPI users taking NSAIDs compared with non‐users. Since PPIs have been used in co‐therapy to prevent the occurrence of NSAIDs‐induced peptic ulcers, and NSAIDs have been shown to have a protective effect on bone when used in combination with PPIs, concurrent NSAID use might have had a confounding influence on bone around dental implants among PPI users. Since the outcome measurement of this study was dental implant failure, the direct incremental effect of PPI on bone loss was not determined. Within the noted limitations, the authors found a relationship between PPIs and dental implant failure.

Chrcanovic et al. (2017), in a retrospective cohort study, studied patients treated with implant‐supported/retained prostheses at a private specialty practice who were or were not taking PPIs. A total of 3559 implants were placed in 999 patients, with 178 implants reported as failures. The implant failure rates were 12% (30/250) for PPI users and 4.5% (148/3,309) for nonusers. The authors concluded that the use of PPIs might be associated with a statistically significant negative effect on the implant survival rate. Although a wide time range resulted in correspondingly large sample size, the variability caused by various clinicians and implant systems used over the course of the study was a study limitation. Additionally, the specific criteria for implant failure were not defined. The outcome of this study was implant failure, so the mechanism through which PPIs affected bone loss could not be identified. That outcome could be observed only when sufficient bone loss had occurred, resulting in loss of osseointegration. Finally, the authors did not perform statistical analysis on differences between PPI users and nonusers at the patient level.

Altay et al. (2019), in a retrospective cohort study involving 1918 dental implants in 592 patients (69 implants in 24 PPI users and 1849 implants in 568 nonusers) evaluated the effect of PPI intake on the osseointegration of dental implants using patient‐ and implant‐level models. There were statistically significant differences between PPI users and nonusers at the implant level, but the authors failed to show any significance at the patient level. The implant failure rates were 4.60 times greater among PPI users versus nonusers. The authors concluded that PPI use might be associated with an increased risk of early dental implant failure. This appeared to be a well‐designed human study that investigated the effects of PPI intake on osseointegration failure at both patient and implant levels. One limitation included a relatively small sample size (24 PPI patients). Second, PPI users received a significantly greater number of implants in the maxilla than in the mandible, which might have influenced the outcome. However, the focus of this study was early dental implant failure, and any direct effect of PPI on bone levels was not determined. The effect of PPIs could be seen only when bone loss exceeded the threshold required to maintain osseointegration, resulting in implant failure.

Ursomanno et al. (2020), in a retrospective record review, assessed the medical and dental histories and radiographic data of 635 patients receiving 1,480 dental implants from 2010 to 2017. The authors found a significantly greater number of exposed threads from the PPI patient group than from PPI nonusers. Patients taking PPIs experienced a 5.5% implant failure rate, while patients not taking such medications had a 2.0% implant failure rate. The strengths of this study included a large patient population with appropriate controls and statistical analysis, with the same examiner responsible for all study‐related radiographic measurements blinded to the patient PPI status. That study was unique since bone loss around implants was quantified by measuring bone loss in millimeters, as well as through quantitation of the number of exposed implant threads, which might serve as a more sensitive assessment of the effect of PPI on implant‐bone level, compared to implant failure. Limitations included the retrospective nature of the study and lack of information on dose and length of time each patient was taking PPIs.

A cross‐sectional study by Romandini et al. (2021), reported on the prevalence, risk factors, and protective indicators of peri‐implant disease. Ninety‐nine patients with 458 dental implants placed between September 2000 to July 2017 at a university clinic were included. Risk/protective indicators were tested individually by using peri‐implantitis as the dependent variable for each potential indicator to test for significance. Using uni‐ and multivariant logistic regression analyses, the authors found peri‐implantitis to be inversely associated with use of PPIs, one of two studies in this scoping review to report an inverse association between PPIs and implant failure. Although a statistically significant association was found between PPIs and peri‐implantitis, the number of implant failures and the number of patients using PPIs was not reported. Furthermore, that report included only implants that had been successfully placed and loaded for a minimum of 1 year. Since early implant failures often occur due to unsuccessful osseointegration or impaired bone healing within the first year if placement (Mohajerani et al., 2017), many implant failures might not have been included in their analysis. This could explain why PPIs were identified as protective indicators for peri‐implant disease.

An abstract by Yerke et al. (2019), described patients with generalized, chronic, moderate‐to‐severe periodontitis using, or not using, PPIs. They found an inverse relationship between the use of PPI and the severity of periodontal disease. The use of PPIs was associated with a decreased proportion of elevated periodontal pocket depths, implying less severe periodontal disease. Strengths included a relatively large sample size (581 patients) with appropriate controls and appropriate statistical analyses.

An abstract by Yerke and Cohen (2019) reported the impact of PPIs on bone levels around teeth in patients with moderate‐to‐severe periodontitis. The authors evaluated patients after adjusting for systemic factors and habits, such as inflammatory bowel disease, smoking, diabetes, use of systemic steroids, peri‐menopausal hormone replacement therapy, hypothyroidism, and another autoimmune disease. Patients using PPIs were associated with reduced percentages of teeth with elevated periodontal probing depths when compared to non‐users. The authors used a comparatively large sample size (518 patients) with controls and statistical analyses. This study expanded upon the previously published abstract to show that a relationship existed between PPIs and decreased probing depths among patients having or not having the potentially confounding conditions noted above. Consequently, the studies described in both abstracts analyzed different subsets of patients that were obtained from the same patient population.

A 2022 cross‐sectional study retrospectively analyzed patients from a university‐based faculty periodontics practice (Chawla et al., 2022). The proportion of probing depths ≥ 5 mm or ≥ 6 mm was measured for patients diagnosed with generalized periodontitis, stage III to IV, grade B to C, among PPI users compared to non‐users. A 31% decrease in probing depths ≥ 5 mm, and a 42% decrease in probing depths ≥ 6 mm, was noted among patients taking PPIs. Those decreases in probing depths remained statistically significant even after excluding smokers and diabetics, as well as patients with systemic disease and taking potentially confounding medications, such as prednisone. In addition, there were no statistically significant differences in oral hygiene between groups, suggesting that plaque control was not a confounding factor. This study had a relatively large sample size (*N* = 1093) and used two probing depth minimum thresholds to assess periodontal severity.

An abstract from the same research group used a similar protocol to evaluate the association between severity of periodontal disease and PPI use among patients seeking care at a private periodontal practice from 2016 to 2019 (Yerke et al., 2021). In a comparatively large population (*N* = 1513), the prevalence of probing depths ≥ 5 mm was 28.1% in PPI users, versus 55.8% in nonusers (95% confidence interval = 14.2%–41.5%, *p* < .001). A similar decrease in the prevalence of probing depths ≥6 mm was observed among PPI users. No differences in plaque control were evident between the PPI and non‐PPI groups.

The proportion of probing depths ≥ 6 mm also was used to assess periodontal severity in another abstract from this group (*N* = 1017) (Herrmann et al., 2022b). The study population included patients seeking care from a university postgraduate periodontics program during 2010–2017. Statistically significant decreases in the proportion of probing depths ≥6mm among non‐PPI users again were noted, with no difference between plaque control between PPI users and nonusers. The studies from this study group were retrospective and therefore not able to account for the dose or duration of PPI use. However, the consistent decrease of periodontal disease severity among PPI users in three different populations suggests that this association is not spurious.

Ursomanno et al. (2019), examined patients receiving dental implants at a university clinic between 2010 and 2017. In an abstract, the authors reported the extent of radiographic bone loss around implants and enumerated the number of exposed threads secondary to crestal bone loss among patients using, or not using, PPIs. The authors concluded that the use of PPIs was associated with greater bone loss around dental implants. Similar results were found after adjusting for confounding factors. A comparatively large sample size (655 patients; 1480 implants) with appropriate controls and statistical analyses were used.

Rogoszinski et al. (2020) evaluated dental implants placed in patients at an unspecified practice setting between 2006 and 2013. The authors evaluated radiographic bone loss, bleeding on probing, suppuration, and periodontal probing depth among patients using, or not using, PPIs. In this study, only dental implants that survived for a minimum of 5 years were considered, yielding 881 implants in 284 patients that were evaluated. In that study, the authors found a 29.7% decreased risk of peri‐implantitis among patients taking PPI medication, which was statistically significant. This publication and Romandini et al. (2021) are the only studies that suggested a protective role for PPIs on bone and tissue attachment levels around dental implants. In any event, one important limitation of the Rogoszinski et al. study was that implants that failed to integrate, or were lost before the five‐year minimum survival criterion, were omitted from their analysis.

A recent study by Rogoszinski et al. (2022) evaluated dental implants at the Philadelphia Veterans Affairs Medical Center during the same time period as his previous study, 2006 to 2013. They evaluated radiographic bone loss, bleeding on probing, suppuration, and periodontal probing depth among those using, or not using, PPIs. Only implants that had a minimum of 5 years of follow‐up were included. The authors assessed the effect of potential confounders, such as smoking, diabetes, age, sex, smoking, implant location, implant type, and whether the site received prior bone grafting. Univariate analysis identified statistically significant associations with confounders, such as smoking, diabetes, and age, with long‐term implant failure and peri‐implantitis. When multivariate analysis was performed, PPI use was not associated with long‐term implant failure or peri‐implantitis. Limitations included the omission of implants lost before 5 years of follow‐up and a lack of information regarding the dose and duration of PPIs. However, this study did require a 5‐year minimum duration of PPI use and required initiation of PPI medication to have occurred before implant placement. A disadvantage is the lack of independence within the implant data: 933 implants were placed in 284 individuals, implying that most individuals had multiple implants that were analyzed separately. Oral hygiene as a risk factor was not assessed.

## 4 DISCUSSION

Collectively, the existing literature suggests a potential relationship between PPIs and tissue attachment around teeth and dental implants. To assess the quality of the literature, we were able to include five full‐length retrospective cohort studies, two cross‐sectional studies, and six abstracts among the 27 publications that initially met the inclusion criteria. Papers that were not further considered for analysis either did not meet the inclusion criteria, or referred to publications that were already individually considered in the current review. There were an insufficient number of studies available to perform a meta‐analysis or systematic review of the effect of PPIs and bone loss around teeth and dental implants. Consequently, a scoping review was performed. Nevertheless, we believe that the current work represents the most comprehensive assessment of the potential effect of PPIs on the periodontium to date, since prior reviews generally were limited to either the effect of PPIs on bone at nonoral sites, or the association of PPIs with dental implant survival. In contrast, the effects of PPIs on bone and soft tissue attachment around teeth and dental implants both were assessed herein. Since not every publication investigating the effect of PPIs on dental implants assessed clinical attachment levels, dental implant failure was used as a surrogate measurement when appropriate, as implant failure implied complete loss of clinical attachment.

The literature describing the relationship of PPIs and teeth was more limited than the relationship between PPIs and implants. We were able to identify five studies describing the effects of PPIs on tissue attachment around teeth, which found an inverse relationship between PPIs and severity of periodontal disease (Chawla et al., 2022; Herrmann et al., 2022b; Yerke & Cohen, 2019; Yerke et al., 2019, 2021). There were no studies that described the effects of PPIs on bone levels around teeth. In contrast, the relationship between PPIs and dental implants appears to be more thoroughly investigated. Results from Aim 2 generally suggest that, with two exceptions, proton pump inhibitors most likely have a negative effect on bone around dental implants, with evidence suggesting a positive relationship between PPIs and bone loss, or no effect (Rogoszinski et al., 2022). There generally was a higher risk of dental implant failure among subjects using PPIs than nonusers, as concluded by Wu et al. (2017), Chrcanovic et al. (2017), Altay et al. (2019), and Ursomanno et al. (2020). That finding is also consistent with a report of more crestal bone loss at implant sites among PPI users, as suggested by Ursomanno et al. (2020). Those five studies appeared to be high‐quality human studies with appropriate sample sizes, controls, and statistical analyses. On the other hand, two studies that reported a protective effect of PPIs on tissue attachment levels around dental implants were unique in that they required a minimum survival period of either 5 years (Rogoszinski et al., 2020, 2022) or 1 year (Romandini et al., 2021) following implant placement as an inclusion criterion. Consequently, those study designs excluded all early implant failures that might or might not have been influenced by PPI use.

Studies using rat models to evaluate the effect of PPIs on dental implant osseointegration have reported both positive and negative effects. A recent study by Tekin et al. (2021) found no difference in biochemical markers (serum alkaline phosphatase, calcium, phosphorous, aspartate aminotransferase, alanine aminotransferase, urea, and creatinine) or biomechanical implant properties (measured in torque value) among animals treated or not treated with omeprazole for 4 weeks. Tekin et al. used a similar study design as a 2016 report by Al Subaie et al. (2016), who demonstrated that omeprazole decreased the bone‐to‐implant contact surface. Since Tekin et al. excluded failed implants in their analysis, the effect of PPIs on early implant failure was not assessed, and the results are influenced by the same limitations as those associated with the Romandini et al. (2021) and Rogoszinski et al. (2020) studies. Moreover, as Tekin et al. (2021) noted in their discussion, tibial bone and maxillary/mandibular bone have different properties and might have different responses to PPIs. Finally, it is unclear whether a 4‐week PPI experimental period is sufficiently long to produce an observable effect. As a result, those study limitations might have contributed to the contradictory results observed by those authors.

Factors that might affect the influence of PPIs on tissue attachment levels around teeth and dental implants, such as medications, systemic conditions (e.g., diabetes), and habits (e.g., smoking) were not individually assessed due to the limited number of publications describing those effects. Another limitation of the present review is English language restriction, which might have decreased the number of relevant publications found in database searches. In addition, this scoping included a discussion of some publications written by the same authors as the present review, which introduces the possibility of potential bias. Nevertheless, this review indicates that additional work is indicated to further elucidate the effect of PPIs on attachment loss. To address this concern, prospective animal and human clinical studies either are planned or in progress in our laboratory.

### 4.1 Possible mechanisms affecting tissue attachment around teeth and dental implants

There are a number of explanations describing how PPIs might influence periodontal and peri‐implant tissues. PPI use has been linked to changes in the gut microbiota (Jackson et al., 2016). Freedberg et al. (2015) found a greater than 10‐fold increase in *Streptococcaceae* in the upper GI tract. An increase in gastric pH can result in a significant increase in oral bacteria, such as *Peptostreptococcus stomatitis, Streptococcus anginosus, Parvimonas micra, Slackia exigua*, and *Dialister pneumosintes* in the GI tract (Bruno et al., 2019). Mishiro et al. (2018) found a significant difference in salivary bacteria when comparing PPI users versus nonusers. As a result, it is conceivable that a shift in microbial diversity with PPI use can affect the number and distribution of periodontopathic organisms, which also might affect the extent or severity of periodontal disease, and thereby affect attachment levels around teeth.

PPIs also have been shown to increase the rate of orthodontic tooth movement, suggesting that an alteration in bone metabolism occurs that can be characterized as an increase in bone resorption relative to bone formation (Makrygiannakis et al., 2018). Similarly, Shirazi et al. (2014), using a rat model system, found a significant increase in optical density of the mandibular bone apical to the first molar after the sixth week of pantoprazole administration, suggesting the development of bone density alterations leading to an increased rate of orthodontic tooth movement, possibly due to decreased calcium absorption.

PPIs affect bone metabolism by impairing intestinal calcium absorption, leading to decreased calcium availability in circulation (Wu et al., 2017). Schinke et al. demonstrated that impaired gastric acidification negatively affects calcium homeostasis, triggering either hyperparathyroidism‐induced bone loss or, when combined with diseases involving osteoclast dysfunction, impaired skeletal mineralization (Schinke et al., 2009). Osteoclasts, like gastric parietal cells, contain acidic vesicles that can be inhibited by PPIs. In vitro studies show that omeprazole inhibits the vacuolar H^+^‐ATPase (V‐ATPase) in bone‐derived membrane vesicles, although at higher levels than those needed for inhibition of gastric H^+^/K^+^ ATPase (Costa‐Rodrigues et al., 2013). The V‐ATPase proton pump is located in the bone‐apposed osteoclast plasma membrane, and proton pump inhibition prevents the secretion of hydrogen ions, decreasing their bone resorption ability (Costa‐Rodrigues et al., 2013). However, PPIs might be a desirable situation in the context of bone tissue disorders, such as periodontal disease, where a decrease in osteoclastic activity might result in a significant improvement in overall bone strength and integrity (Costa‐Rodrigues et al., 2013). That possibility was proposed by Yerke et al. (2019) who found an inverse relationship between use of PPI and severity of periodontal disease.

Some studies have reported a two‐ to four‐fold increased risk of B_12_ deficiency associated with PPI therapy (Freedberg et al., 2017). By altering intragastric pH levels, PPIs decrease the absorption of vitamin B_12_. Vitamin B_12_ is an essential protein‐bound nutrient where the presence of gastric acid is required for pancreatic proteases to cleave vitamin B_12_ from protein, allowing its reassociation with intrinsic factor and eventual absorption in the terminal ileum (Ito & Jensen, 2010). Short‐term studies have suggested that acid suppressants such as PPIs decrease the absorption of vitamin B_12_ (Makrygiannakis et al., 2018). A few cross‐sectional studies have shown low serum vitamin B_12_ levels to be associated with decreased levels of markers of bone formation (Stone et al., 2004). Therefore, it is possible that PPIs might have a potential negative effect on tissue attachment around teeth or dental implants by decreasing B_12_ availability.

Chronic iron deficiency has been suggested to negatively affect bone (Toxqui & Vaquero, 2015). Dietary iron is present in food as either nonheme (66%) or heme iron (32%), with absorption of nonheme iron being markedly improved by gastric acid. Gastric acid facilitates the dissociation of nutrients from iron salts, which allows the formation of complexes with ascorbate, sugars, and amines. Numerous clinical conditions associated with achlorhydria/hypochlorhydria have been shown to be associated with decreased iron absorption and/or iron‐deficiency anemia (Ito & Jensen, 2010). Sarzynski et al. (2011) in a retrospective cohort study, found significantly decreased hematologic indices, including hemoglobin amongst PPI users when compared with matched controls (Sarzynski et al., 2011). Therefore, PPI‐induced iron deficiency might have a deleterious effect on bone and tissue attachment around teeth or dental implants.

Increasing evidence suggests that PPIs might disrupt skeletal integrity through PPI‐induced hypochlorhydria, which impairs calcium absorption and reduces calcium bioavailability for incorporation into bone, resulting in compensatory and potentially chronic hyperparathyroidism that increases bone turnover (Hinson et al., 2015). Hinson et al. (2015) in a retrospective chart review of individuals 60 years and older, found statistically significant and pathologically higher PTH levels among chronic PPI users. The persistent elevation of PTH levels might lead to loss of bone strength and quality. Collectively, those findings might provide additional insight regarding the mechanism through which PPIs influence bone metabolism around teeth or implants.

The present results also have applicability to clinical practice. Alternative drugs to PPIs might be explored for patients at a higher risk for dental implant failure (Wu et al., 2017). Histamine 2‐receptor blocker or over‐the‐counter antacids could be considered before prescribing the most potent available treatment (Benmassaoud et al., 2016). Mitigation of potential PPI risks could be attempted by PPI reduction or by giving risk‐specific supplements (Freedberg et al., 2017). However, the literature regarding the use of supplements to ameliorate potential PPI risks also is limited (Freedberg et al., 2017). Probiotics show a modest benefit in preventing antibiotic‐associated diarrhea but have not been tested to prevent infections in long‐term users of PPIs (Freedberg et al., 2017). Supplementation of calcium and vitamin D does not conclusively decrease risk for fracture (Freedberg et al., 2017). Indeed, it has been recommended that patients taking PPIs should not use them for indefinite period, but rather attempt to taper once symptoms are controlled (Benmassaoud et al., 2016). Since there appears to be an association between PPI use and implant failure, dentists might consider PPIs to be a risk factor when planning an implant surgery for their patients. Consequently, clinicians should consider whether more frequent maintenance visits and home care reinforcements might be indicated for implant patients taking PPIs.

On the other hand, preliminary results suggest that PPIs appear to be beneficial for tissue attachment around teeth and could potentially be prescribed short‐term, for patients at a risk for periodontitis. However, further well‐controlled prospective studies will be required to corroborate the relationship between PPIs and tissue attachment around teeth before making treatment recommendations regarding adjunctive use of PPI in periodontal therapy. The precise mechanisms through which PPIs affect the supporting structures around teeth or dental implants also remain to be fully elucidated.

## 5 CONCLUSIONS

There are few high‐quality studies describing the potential association between PPIs, bone, and tissue attachment, and teeth and dental implants, with most of the available literature consisting of retrospective cohort and chart reviews. In general, and among the included papers with the fewest confounding factors, PPIs appear to have a predominantly negative but variable effect on peri‐implant tissues and might exert a potentially protective effect on periodontal tissues.

## AUTHOR CONTRIBUTIONS

Lisa M. Yerke and Robert E. Cohen supervised the study and were responsible for all manuscript revisions. Bhavneet K. Chawla was responsible for the first draft. Elizabeth M. Stellrecht performed the literature search and verified that the scoping review followed PRISMA guidelines. All authors reviewed and approved the final version.

## CONFLICT OF INTEREST

The authors declare no conflict of interest.

## Data Availability

All data available in article. If there are questions, the corresponding author can be contacted.
